# Vitamin D Receptor—Interplay in COVID-19-Negative, -Infected, and -Vaccinated Women during Pregnancy

**DOI:** 10.3390/jcm13206140

**Published:** 2024-10-15

**Authors:** Constantin Condac, Ludmila Lozneanu, Daniela Roxana Matasariu, Alexandra Ursache, Iuliana Elena Bujor, Maria Elena Niță, Vasile Lucian Boiculese, Mihai Sava, Paula Țăroi, Victoria Bîrluțiu

**Affiliations:** 1 Department of Anesthesia and Intensive Care, “Cuza Vodă” Hospital, 700038 Iasi, Romania; costicondac@gmail.com; 2 Department of Infectious Diseases, University of Medicine and Pharmacy “Lucian Blaga”, 550169 Sibiu, Romania; victoria.birlutiu@ulbsibiu.ro; 3 Department of Morpho-Functional Sciences I—Histology, University of Medicine and Pharmacy “Gr. T. Popa”, 700115 Iasi, Romania; ludmila.lozneanu@umfiasi.ro; 4 Department of Obstetrics and Gynecology, University of Medicine and Pharmacy “Gr. T. Popa”, 700115 Iasi, Romania; iuliana-elena.bujor@d.umfiasi.ro; 5 Department of Obstetrics and Gynecology, “Cuza Vodă” Hospital, 700038 Iasi, Romania; elemarni28@gmail.com; 6 Biostatistics, Department of Preventive Medicine and Interdisciplinarity, University of Medicine and Pharmacy “Gr. T. Popa”, 700115 Iasi, Romania; lboiculese@gmail.com; 7 Department of Anesthesia and Intensive Care, University of Medicine and Pharmacy “Lucian Blaga”, 550169 Sibiu, Romania; mihai.sava@ulbsibiu.ro; 8 Department of Obstetrics and Gynecology, University of Medicine and Pharmacy “Lucian Blaga”, 550169 Sibiu, Romania; paula.nita@ulbsibiu.ro

**Keywords:** placenta, SARS-CoV-2, COVID-19, vitamin D receptor, immunohistochemistry, diagnosis, Pfizer vaccine

## Abstract

**Background:** The trophoblast is a significant source of vitamin D synthesis during pregnancy, with the literature suggesting its role in fetal growth. We aim to underline a possible mechanism that would explain negative fetal outcomes in COVID-19-positive mothers by examining the relationship between altered placental structure and function and throphoblast cells‘ vitamin D receptor levels. **Methods:** The study included 170 placental samples collected from women who gave birth at term without complications, divided into three groups: COVID-19-positive and unvaccinated, COVID-19-negative and vaccinated, and COVID-19-negative and unvaccinated, with exclusion criteria for any other medical complications. Immunohistochemistry (IHC) was performed to detect vitamin D receptor (VDR) expression, and immediate fetal outcomes (weight and Apgar score) were assessed. **Results:** We found lower gestational age at birth, lower birth weight, and reduced placental VDR (vitamin D receptor) levels in COVID-19-positive women compared to COVID-19-vaccinated and COVID-19-negative women. **Conclusions:** The presence of the vitamin D receptor in the placenta is related to fetal and placental growth. Its deficiency may contribute to negative fetal outcomes in COVID-19-positive cases.

## 1. Introduction

Coronavirus disease 2019 (COVID-19) is an acute respiratory illness caused by SARS-CoV-2 infection [1,2]. COVID-19 infection can negatively impact various organs and tissues, leading to diverse clinical manifestations [3]. Beyond respiratory symptoms, it can impact gastrointestinal function; cause preeclampsia in pregnant women; affect liver function; impair renal, cardiac, and male fertility function; and cause neurological, immune, and endocrine system dysfunction [4,5,6]. The virus accesses human cells via the angiotensin-converting enzyme 2 (ACE-2), which is abundantly present on the placental syncytiotrophoblast, suggesting a possible connection between infection and impaired placental function [7,8].

The impact of COVID-19 on pregnant women and infants is of particular interest to obstetricians, pediatricians, and patients [9]. Pregnancy increases the vulnerability to severe forms of COVID-19 due to physiological changes, particularly in the cardiovascular and immune systems. However, its impact on maternal and fetal outcomes remains a topic of debate. There is evidence linking COVID-19 in pregnancy to complications such as hypertensive disorders, preterm birth, fetal growth pathology, gestational diabetes, increased neonatal intensive care admission, and stillbirth. Discrepancies in the literature are further pronounced when considering the effects of different viral strains and the outcomes for asymptomatic or mildly symptomatic pregnant women. There is also persistent controversy about the vertical transmission of COVID-19 infection, either mediated by angiotensin-converting enzyme 2, or through hypoxemia-injured placenta [10,11]. The pathological analysis of placenta during past epidemics has been informative regarding the resulting negative outcomes and the mechanisms of disease transmission to the fetus. Extensive research has been conducted to explain the physiological and pathological mechanisms of COVID-19, to limit its spread and consequences [12,13,14].

Vitamin D plays multiple roles in the human organism, including the development of the reproductive axis, homeostasis, bone formation, and the modulation of both innate and adaptive immune systems. It supports placental development and function by regulating calcium transport, which is essential for pregnancy maintenance, and preventing a maternal immune response against the embryo, playing a pivotal role in fetal growth and development [15,16,17]. The literature is abundant in studies that relate low vitamin D serum levels to the severity of the COVID-19 infection, underlining vitamin D’s well-known role in modulating the immune response, depending on the expression of its receptor [17,18,19,20]. From the first trimester of pregnancy, the placental levels of vitamin D receptor (VDR) increase significantly, underlining vitamin D’s importance in fetal development and placental physiology, with some authors suggesting that it shows antimicrobial and anti-inflammatory activity [21].

The available literature contains limited data on vitamin D levels in COVID-19-positive pregnant women [20,22], with only one study by Moreno-Fernandez et al. evaluating placental vitamin D levels [23], which prompted our study to try to clarify the connection between COVID-19 infection and adverse obstetrical outcomes. We aimed to underline a possible mechanism that would explain negative fetal outcomes in COVID-19-positive mothers by examining the relationship between altered placental structure and function and trophoblast cells’ vitamin D receptor levels.

## 2. Materials and Methods

### 2.1. Patients and Tissue Samples

The study period was from January 2021 to January 2023. All of the patients provided written informed consent. The study was approved by the ethics committees of the University of Medicine and Pharmacy “Lucian Blaga”, Sibiu (1442/19 March 2024) and the Obstetrics and Gynecology Hospital “Cuza-Voda” in Iasi, Romania (No. 10426/24 August 2021 and No. 19/4 August 2023).

Specimens were collected from women who gave birth at term without complications, associated disease, or chronic treatment (for toxoplasma; rubella; cytomegalovirus; herpes with no acute infection detected; hepatitis B or C; HIV; or syphilis).

The pregnant women were divided into three groups: COVID-19-positive and unvaccinated women; COVID-19-negative and vaccinated women; and COVID-19-negative and unvaccinated women. Group 1, comprising the COVID-19-positive and unvaccinated women, included asymptomatic patients, with the disease detected at any time during pregnancy but at least 14 days before birth. The diagnosis of COVID-19 positivity was confirmed using Polymerase Chain Reaction (PCR) testing. The second group included Pfizer-vaccinated women, with the first or second dose administered at least 14 days before birth, who never tested positive for COVID-19 during the pandemic and achieved negative results for all their rapid COVID-19 tests, which were taken every three weeks during pregnancy. The control group included COVID-19-negative and unvaccinated women who gave birth at term without any complications or associated pathology (all the women included in the third group never tested positive during the pandemic until inclusion in our study, and had returned negative rapid COVID-19 tests every three weeks during pregnancy).

#### Exclusion Criteria

Women with obstetrical and/or other medical complications were excluded from our analysis. Patients with COVID-19 infection at the time of giving birth; malignancy; depression; genetic syndromes; infectious or autoimmune diseases; pre-existing or gestational diabetes; hypertension and its complications, such as preeclampsia; premature rupture of membranes; oligohydramnios; intrauterine growth restriction, defined as ultrasound-estimated fetal weight less than the 10th percentile for gestational age; or chorioamnionitis and those who smoked were excluded.

### 2.2. Immunohistochemistry

We collected four tissue samples from each placenta, one for each of the four quadrants. Hematoxylin and eosin (H&E) sections were examined, and immunohistochemistry (IHC) was evaluated by two pathologists. IHC staining was performed on formalin-fixed, paraffin-embedded tissues for immunohistochemistry utilizing monoclonal antibodies against VDR. Four-micrometer-thick serial sections were prepared in citrate buffer (pH 6) after deparaffinization in xylene and rehydration in ethanol series. Endogenous peroxidase activity was inhibited with 0.3% H_2_O_2_ for 20 min at room temperature. IHC was used to determine the expression of vitamin D receptor (VDR) that was diluted at 1:3000 (catalog no ab3508 Abcam, Cambridge, UK) and incubated overnight at 4 °C. The sections were washed, exposed to the secondary antibody for 45 min at 37 °C, and cleaned with phosphate-buffered saline (PBS). Hematoxylin was used as a counterstain in the standard avidin-biotin-peroxidase technique, using a liquid DAB (diaminobenzidine) substrate and chromogen system. Human jejunum tissue was used as a positive control.

One hundred and seventy placental samples were examined for VDR presence. Positive cells (with a brown or yellowish-brown color in the nucleus) in epithelial and stromal compartments were considered VDR-positive, regardless of the intensity of staining or the number of positive cells.

### 2.3. Statistical Analysis

Data were imported into Microsoft Excel and analyzed in SPSS 24 (IBM Corp. Released 2016. IBM SPSS Statistics for Windows, Version 24.0. Armonk, NY, USA: IBM Corp.). Descriptive statistics included sample size (N), mean, standard deviation, standard error, 95% confidence interval, minimum, maximum, and absolute and relative frequencies. Non-parametric statistical hypothesis tests, such as the Kruskal–Wallis test for 3 samples and post hoc tests using Bonferroni–Dunn corrections for 2-sample comparisons, were applied. These have been applied to the analysis of continuous numerical variables. Chi-square or Fisher exact tests were applied for categorical variables. A significance level of 0.05 was considered statistically significant.

Since we had small sample volumes, we performed a statistical power analysis using the G*power 3.1.9.7 application. Post hoc power analysis was conducted based on the sample sizes, the effect, and the type I error rate of α = 0.05. We simulated different scenarios for real continuous variable hypotheses tests using the Kruskal–Wellis test, and also for frequency comparisons with chi-square distribution. The power 1-β ranges between 0.09 and 0.93.

## 3. Results

After applying the inclusion and the exclusion criteria, 170 pregnant women were included in the study: 65 unvaccinated COVID-19-positive women in group 1, 35 vaccinated and COVID-19-negative women in group 2, and 70 unvaccinated COVID-19-negative women in group 3. All of the COVID-19-positive patients in the study were asymptomatic. The ages of our patients ranged from 18 to 39 years.

All of the study patients were asymptomatic. None required supplemental oxygen administration or admission to the intensive care unit. Laboratory testing, involving complete blood counts, inflammatory markers, and biochemical profiles, was performed. Neonatal outcomes, including birth weights, APGAR scores, and neonatal intensive care unit (NICU) admissions, were similar between the three groups (Table 1).

The unvaccinated pregnant women had a mean age that was 3 years lower than that of the vaccinated women. When we analyzed our groups based on their urban or rural provenance, we detected that in the COVID-19-positive group, 73% of the included cases had urban residency, as did almost 69% from the vaccinated group, while in the COVID-19-negative unvaccinated group, the distribution by residency was equal.

A significant positive correlation was observed between birth gestational age and birth weight. The mean gestational delivery age in our three groups showed significant differences: COVID-19-positive and COVID-19-vaccinated pregnant women had a lower birth gestational age compared to the COVID-19-negative unvaccinated group (Table 2). Another statistically significant difference between the three groups became evident when analyzing the birth weights of the newborns, with children from the COVID-19-negative and unvaccinated group having a higher birth weight compared to those born to vaccinated or COVID-19-positive pregnant women. However, when we analyzed the immediate after-birth statuses of the newborns as reflected by the Apgar scores, we found no significant difference between the three groups (Table 2).

The serum infection/inflammation markers showed no statistical difference in CRP values among the pregnant participants. However, pregnant women who were neither infected with the virus nor vaccinated had significantly higher leucocyte values (Table 2).

**Figure 1:**
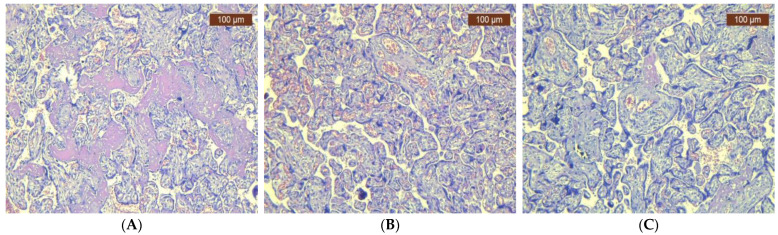
Representative histopathological changes in placenta (H&E). (**A**) COVID-19-positive pregnant women: small, well-vascularized chorionic villi. Syncytial knots and intervillous fibrin (HE × 10). (**B**) COVID-19-vaccinated pregnant women: chorionic villi, congestion, and fibrosis (HE × 20). (**C**) COVID-19-negative and unvaccinated pregnant women: different sizes of chorionic villi, congestion, and area of fibrosis (HE × 10).

When examining H&E sections, pathological changes were depicted. In COVID-19-positive and vaccinated women, we observed signs of inflammation, such as villitis or intervillositis, increased fibrin deposition, and possibly microthrombi. These changes indicate an immune response or vascular involvement associated with the virus. Conversely, in non-infected and unvaccinated women, the placenta showed typical histological features without these specific inflammatory or vascular alterations (Figure 1A–C).

**Figure 2:**
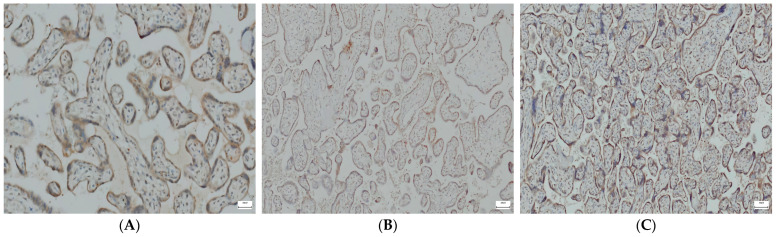
Representative images—immunohistochemical visualization of VDR in mononuclear cells of trophoblasts. (**A**) COVID-19-positive pregnant women (×20). (**B**) COVID-19-vaccinated pregnant women (×20). (**C**) COVID-19-negative and unvaccinated pregnant women (×10).

IHC analysis revealed differences in VDR expression among the three groups, with a statistically significantly higher number of VDR-negative cases in tissue samples originating from the placenta of COVID-19-vaccinated women (*p* < 0.001). The number of VDR-positive cases was higher in non-vaccinated COVID-19-negative pregnant women compared to COVID-19-positive and vaccinated ones (Figure 2A–C and Table 3).

## 4. Discussion

The COVID-19 pandemic had a profound socio-economic impact, resulting in significant alterations to daily behavior and routine. It emphasized depression, underlining the importance of assessing personality traits in patients for more effective health management. The healthcare system was particularly strained, with increasing reports of heightened patient admissions and hospitalizations, largely due to reduced outpatient services in both gynecology and obstetrics departments. In gynecology, the most common presenting complaint was lower abdominal pain, alongside oncological emergencies. In addition to the psychological stress associated with gynecological conditions, women were further burdened by concerns over potential contagion. Similarly, obstetrics departments saw a rise in emergency cases, accompanied by a decline in non-urgent cases, likely driven by fears of infection [23,24,25].

A classification system for maternal, fetal, and neonatal COVID-19 infections was proposed by Shah et al. [26]. According to this classification, virus detection by PCR or electron microscopy using fetal or placental tissue, as well as the definitive or probable maternal status, defines a confirmed congenital infection [26,27]. The placenta often has a protective role, inhibiting the transmission of infectious agents from the mother to the fetus [1]. Diverse maternal conditions, including morphological changes, can influence the placenta’s function. For example, tissue hypoxia, a common adverse effect of COVID-19 that is caused by a hypercoagulable state, together with anemia (an independent risk factor), can lower the physiological capacity for oxygen transport when faced with increased demand, such as in placental tissue [28]. Although there were no statistically significant differences in hemoglobin and hematocrit levels among our three groups, the overall incidence of anemia remains high in our region’s pregnant population. Therefore, we may assume that anemia, as an independent risk factor, uniformly impacts all three included groups.

The immunocompromised state of pregnancy makes patients more susceptible to viral respiratory infections, due to the attenuated immune response of the placenta. Hypoxia, inflammatory activation, increased thrombotic events—secondary to COVID-19—due to turbulent and slow blood flow, progressive rises in fibrin degradation products, and decreased fibrinolysis may lead to adverse pregnancy outcomes due to pathological insult of the placenta, whether acute or chronic (such as miscarriage, oligohydramnios, fetal growth restriction, silent placental abruption, postpartum hemorrhage, unexplained stillbirth, lower Apgar score, congenital viral syndrome, birth defect, preeclampsia, preterm birth, cesarean section) [8,14,29,30,31,32,33]. These placental changes can be highly unpredictable and subtle, varying with the severity of the disease and with the viral subtype, with early variants of COVID-19 registering a more significant negative impact on maternal and fetal outcomes compared to later viral subtypes, such as Omicron, making early detection and intervention extremely challenging [32]. Therefore, vigilant antenatal and intrapartum monitoring is essential in such cases [29,32], especially because many pregnant women remain reluctant to undergo vaccination [34].

Our analysis did not reveal any alterations in newborn status besides a lower birth weight in COVID-19-positive and vaccinated mothers, similar to results described in the literature [31,32]. Lymphopenia with a higher neutrophil count has been associated with COVID-19 infection in pregnant women compared to non-pregnant ones [35]. Our study results reflected quite the opposite, with similar CRP levels among our three groups and higher leucocyte values in the COVID-19-negative and unvaccinated groups. The source of this inconsistency might be the low number of cases included in our research, due to stringent inclusion and exclusion criteria, or perhaps due to some regional particularities.

The neonatal outcomes described were Apgar score, fetal birth weight, the need for neonatal intensive care admission, and fetal demise. Immediately after birth, the Apgar scores of and the three days spent in hospital by the newborns from our included cases did not reveal any negative impact of the infection or vaccination on the newborns. No fetal demise was detected, and there was no need for intensive care admission in our cases. Despite the lack of negative impact on our newborns, the infection and multi-organ fetal inflammation (including lung and kidney) produced by COVID-19 infection during early pregnancy, described in the literature, should alert clinicians involved in the assessment and management of pregnant women to possible fetal consequences and adverse perinatal outcomes [27,36]. Even with a normal short-term outcome, long-term sequelae, such as consequences for the neurodevelopment of newborns, cannot be excluded [30,33].

Compared to placentas from non-infected unvaccinated pregnant women, there were no significant differences in macroscopic and microscopic placental appearance, including placental weight, abnormal cord insertion, and maternal or fetal vascular malperfusion. However, because the majority of our analyzed placentas were not infected and the majority of neonates were negative for coronavirus infection, our findings largely came from examining healthy placentas from uninfected fetuses, similar to those obtained by Schwartz et al. [12]. Many authors have reported no definitive vertical transmission of COVID-19, but the findings in the literature are inconsistent [1,3,37,38,39]. Additionally, limited positive cases have been reported (mostly case reports or small case series) in the late pregnancy stage with possible postpartum infections, with a rate of transmission ranging from 0% to 9% [30]. Given the extensive literature supporting the benefits of vitamin D supplementation in COVID-19-positive patients, particularly pregnant women, and the well-documented adverse maternal and fetal outcomes associated with low vitamin D serum levels, we sought to evaluate vitamin D status in such cases [40,41,42]. Assessing serum vitamin D levels in a standardized manner poses serious challenges, as numerous studies report conflicting results due to variations in quantification methods and the wide range of factors influencing vitamin D levels [19,42]. To avoid these limitations, we focused on detecting VDR expression in placental tissue, which may prove to be a more accurate assessment of the impact of COVID-19 infection and vaccination on the levels of vitamin D, an essential molecule with a critical role in placentation and fetal well-being [40,41,42,43]. Since both vitamin D and VDR levels fluctuate throughout pregnancy [19,40,41,42,43], we specifically selected placentas only from women in their third trimester of pregnancy for our IHC analysis.

To the best of our knowledge, apart from a single study by Moreno-Fernandez et al., which evaluated placental vitamin D levels [22], this is the first study to assess VDR expression in placental tissue from COVID-19-positive, COVID-19-negative but Pfizer-vaccinated, and COVID-19-negative and unvaccinated pregnant women. By focusing on VDR rather than serum vitamin D, our study offers a novel approach to understanding how COVID-19 infection and vaccination influence vitamin D pathways during pregnancy, with potential implications for maternal and fetal health. Our results further support the well-established fact that vitamin D supplementation is required in COVID-19-positive cases that exhibit a depletion of this essential compound. Also, the fact that both COVID-19-positive and Pfizer-vaccinated pregnant women revealed lower levels of placental VDR supports this observation even further. Vitamin D supplementation increases VDR expression [28,40]. Thus, vitamin D supplementation might prove to be useful both during COVID-19 infection and also after vaccination, with a potential to improve maternal and fetal outcomes.

The trophoblast is a significant source of vitamin D synthesis in pregnancy, with the literature proving its role in fetal growth [40,41,43]. Vitamin D is involved in a wide range of placental processes, being responsible for immune modulation and impacting hormonal secretion at this level. Its receptor’s presence is also related to immune reactions, as well as fetal and placental growth [40,41,43]. Therefore, our two main findings seem to be related, with the lack of VDR expression impacting fetal growth, although in a non-statistically significant manner. It is important to differentiate the effects of COVID-19 from causes of maternal and fetal malperfusion, such as preexisting pregnancy-induced hypertension and disseminated intravascular coagulation during pregnancy [8], twin pregnancy, smoking, and preeclampsia [19,43]. That is one of the main reasons for our small number of cases—we used very strict inclusion and exclusion criteria to ensure the accuracy of this study’s results.

According to Joshi et al., the placental abnormalities in their study were not related to the symptoms or severity of COVID-19, with no correlation between maternal and fetal disease characteristics [29]. Even with a normal fetal and maternal outcome, most of the placentas (82.6%) presented signs of maternal vascular malperfusion, with features of fetal vascular malperfusion in almost half of the cases [29]. This aspect underlines once more the importance of studying the placenta’s structure and function in viral infections for a better understanding of their exact impact. Despite the fact that COVID-19 infection shares similarities with Zika infection, without the same catastrophic fetal malformation effect, its frequent silent and subtle alteration of the maternal–fetal unit might not be minor at all. It is important to mention that we did not find any maternal symptoms or clinical details that could predict abnormal placental findings in pregnancy complicated by COVID-19 infection [44]. The duration of COVID-19 infection is approximately 20 days, ranging from 5 to 60 days, so the virus may reach and affect the placenta before delivery. This means that the virus can be cleared and no longer be detectable in a placental specimen obtained at childbirth [45]. On the other hand, infection in the late stage of gestation may indicate no evidence of vertical trans-placental COVID-19 transmission, without significant impact on the perinatal outcomes of newborns, in both mild and severe cases [1]. Another important aspect to consider is that these studies included a heterogeneous population for the timing of infection. A small series has reported that in cases of adverse outcomes, a specific placentitis—characterized by trophoblast degeneration, intervillositis, and massive perivillous fibrinoid deposits—has been identified, with similar findings observed in our cases [30].

The limitations of the above-mentioned studies include limited sample sizes and the fact that the asymptomatic forms of COVID-19 included cases with no mild, moderate, or severe disease-affected patients. Also, our group sizes were unequal, with the imbalance resulting in possible bias and impaired generalizability of the results. Our study only focused on short-term fetal outcomes, with limited understanding of the long-term impact of COVID-19 infection on children born from COVID-19-infected asymptomatic mothers. As this is a single-center study, our findings may not be fully representative of other populations from other regions, highlighting the need for further research using a larger cohort to better understand the exact pathophysiological mechanisms behind impaired fetal growth and adverse pregnancy outcomes. Such insights could improve pregnancy management in future epidemics. Another limitation is that we did not analyze or subdivide our COVID-19-infected group based on their gestational age at the time of infection. We plan to expand our research in this area to determine whether specific outcomes and VDR expression are influenced by the timing of the infection during pregnancy, or by disease severity. Also, we want to try to correlate vitamin D serum levels with placental VDR expression and pregnancy outcomes in COVID-19-positive, vaccinated, and control groups, to obtain more insights into the implications of vitamin D in pregnancy, in the context of COVID-19 infection. We also evaluated additional placental markers related to immune response, structure, and function in another study we performed on vaccinated COVID-19-positive and unvaccinated COVID-19-negative women, to explore potential pathways and provide explanations for the increased maternal and fetal morbidity observed during the pandemic.

## 5. Conclusions

Although we detected lower birth weights in COVID-19-positive and COVID-19-vaccinated pregnant women compared to the control group, the difference was not statistically significant. However, we uncovered a higher expression of VDR in placental tissue from unvaccinated COVID-19-negative cases compared to the other two groups in our study. These two findings seem to be connected, serving as a starting point for future pregnancy management during epidemics. The presence of the vitamin D receptor in the placenta is related to fetal and placental growth. Its deficiency may contribute to negative maternal and fetal outcomes in COVID-19-positive cases, suggesting that vitamin D supplementation could help mitigate the negative effects of viral infection.

## Figures and Tables

**Table 1:** Distribution of included cases.

Group	Frequency	Percent	Valid Percent	Cumulative Percent
COVID-19-positive pregnant women	65	38.2	38.2	38.2
COVID-19-vaccinated pregnant women	35	20.6	20.6	58.8
COVID-19-negative and unvaccinated pregnant women	70	41.2	41.2	100
Total	170	100	100	

**Table 2:** Clinical and demographic characteristics of included women.

Clinical and Demographic Characteristics of Women		No	Mean	St. Dev.	Percentile 25	Median	Percentile 75	Min	Max	Kruskal–Wallis *p*-Value
Maternal age (years)	Negative and unvaccinated	70	28	4	27	29	31	19	34	<0.001
	Positive	65	31	5	28	33	34	18	39	
	Pfizer-vaccinated	35	30	5	27	30	33	18	39	
Gestational age at delivery (weeks)	Negative and unvaccinated	70	40	1	38	40	41	37	41	<0.001
	Positive	65	38	3	37	38	39	28	40	
	Pfizer-vaccinated	35	38	3	37	39	40	29	41	
Fetal weight (grams)	Negative and unvaccinated	70	3507	354	3260	3495	3670	2800	4490	0.01
	Positive	65	3237	536	2920	3300	3600	1980	4540	
	Pfizer-vaccinated	35	3291	522	2920	3390	3650	1990	4570	
Apgar score at 1 min (point)	Negative and unvaccinated	70	9	1	8	9	9	8	9	0.67
	Positive	65	8	1	8	9	9	4	9	
	Pfizer-vaccinated	35	8	1	8	9	9	4	10	
Hemoglobin antepartum (milligrams/deciliter)	Negative and unvaccinated	70	12.3	1.1	11.8	12.1	13.6	10.0	13.7	0.76
	Positive	65	12.2	0.8	11.9	12.3	12.5	9.7	13.4	
	Pfizer-vaccinated	35	12.3	0.9	11.9	12.3	13.0	9.8	14.3	
Hematocrit antepartum (%)	Negative and unvaccinated	70	36.3	3.4	35.0	37.0	38.9	28.1	40.0	0.578
	Positive	65	36.4	2.6	35.3	36.0	38.0	29.3	41.0	
	Pfizer-vaccinated	35	36.8	2.9	35.3	36.9	38.8	28.1	42.1	
Hemoglobin postpartum(milligrams/deciliter)	Negative and unvaccinated	70	10.9	0.8	10.4	10.9	11.4	9.0	13.0	0.61
	Positive	65	11.0	0.9	10.4	11.0	11.6	8.9	12.8	
	Pfizer-vaccinated	35	11.1	0.8	10.5	11.0	11.6	9.7	12.9	
Hematocrite postpartum (%)	Negative and unvaccinated	70	31.8	2.9	29.0	31.1	34.3	27.8	37.0	0.52
	Positive	65	32.3	2.8	30.4	32.3	33.6	27.7	38.5	
	Pfizer-vaccinated	35	32.4	2.8	29.9	32.5	34.3	27.5	37.5	
Leucocyte value (10^3^/liter)	Negative and unvaccinated	70	12,293	2272	10,350	11,485	14,600	9900	16,500	<0.001
	Positive	65	10,186	2590	8280	10,120	12,100	5390	16,400	
	Pfizer-vaccinated	35	10,936	2922	8320	10,350	12,280	5390	16,500	
Platelet value (10^6^/liter)	Negative and unvaccinated	70	220,500	78,240	149,000	217,500	278,000	122,000	355,000	0.76
	Positive	65	218,523	63,791	156,000	211,000	260,000	132,000	355,000	
	Pfizer-vaccinated	35	227,543	71,222	156,000	218,000	278,000	135,000	36,0000	
CRP (C-reactive protein) value (milligrams/deciliter)	Negative and unvaccinated	70	3.12	1.52	2.00	2.75	4.10	0.90	10.00	0.89
	Positive	65	3.09	1.30	1.90	3.00	4.80	1.07	4.90	
	Pfizer-vaccinated	35	3.18	1.20	2.30	2.80	4.10	1.00	4.90	

**Table 3:** VDR status in our three groups of pregnant women.

Group		VDR		Total	*p*-Value
		Negative	Positive		
COVID-19-positive pregnant women	Count	29	36	65	0.044
	% within group	44.6%	55.4%	100.0%	COVID-positive vs. COVID-vaccinated
Pfizer-vaccinated pregnant women	Count	23	12	35	<0.001
	% within group	65.7%	34.3%	100.0%	COVID-vaccinated vs. control
COVID-19-negative and unvaccinated pregnant women	Count	14	56	70	0.002
	% within group	20.0%	80.0%	100.0%	COVID-positive vs. control
Total	Count	66	104	170	
	% within group	38.8%	61.2%	100.0%	

## Data Availability

The data used to support the findings of this study are available upon request to the corresponding author.
